# Exploiting mitochondrial and metabolic homeostasis as a vulnerability in NF1 deficient cells

**DOI:** 10.18632/oncotarget.19335

**Published:** 2017-07-18

**Authors:** Robert J. Allaway, Matthew D. Wood, Sondra L. Downey, Stephanie J. Bouley, Nicole A. Traphagen, Jason D. Wells, Jaya Batra, Sir Norman Melancon, Carol Ringelberg, William Seibel, Nancy Ratner, Yolanda Sanchez

**Affiliations:** ^1^ Department of Molecular and Systems Biology, Geisel School of Medicine, Dartmouth College, Hanover, NH 03755, USA; ^2^ Department of Pharmacology and Toxicology, Geisel School of Medicine, Dartmouth College, Hanover, NH 03755, USA; ^3^ Department of Epidemiology, Geisel School of Medicine, Dartmouth College, Hanover, NH 03755, USA; ^4^ Bioinformatics Shared Resource, Norris Cotton Cancer Center, Dartmouth-Hitchcock Medical Center, Lebanon, NH 03756, USA; ^5^ Division of Oncology, Cincinnati Children’s Hospital Medical Center, Cancer and Blood Diseases Institute, Cincinnati, OH 45229, USA; ^6^ Division of Experimental Hematology and Cancer Biology, Cincinnati Children’s Hospital Medical Center, Cancer and Blood Diseases Institute, Cincinnati, OH 45229, USA; ^7^ Norris Cotton Cancer Center, Dartmouth-Hitchcock Medical Center, Lebanon, NH 03756, USA; ^8^ Current address: Department of Pathology, University of California San Francisco, San Francisco, CA 94143, USA; ^9^ Current address: Icahn School of Medicine at Mount Sinai, New York, NY 10029, USA; ^10^ Current address: Vanderbilt School of Medicine, Nashville, TN 37232, USA

**Keywords:** neurofibromin 1, RAS, synthetic lethal, mitochondria, proteostasis

## Abstract

Neurofibromatosis type 1 is a disease caused by mutation of neurofibromin 1 (*NF1*), loss of which results in hyperactive Ras signaling and a concomitant increase in cell proliferation and survival. Patients with neurofibromatosis type 1 frequently develop tumors such as plexiform neurofibromas and malignant peripheral nerve sheath tumors. Mutation of *NF1* or loss of the NF1 protein is also observed in glioblastoma, lung adenocarcinoma, and ovarian cancer among other sporadic cancers. A therapy that selectively targets NF1 deficient tumors would substantially advance our ability to treat these malignancies.

To address the need for these therapeutics, we developed and conducted a synthetic lethality screen to discover molecules that target yeast lacking the homolog of *NF1*, *IRA2*. One of the lead candidates that was observed to be synthetic lethal with *ira2Δ* yeast is Y100. Here, we describe the mechanisms by which Y100 targets *ira2Δ* yeast and NF1-deficient tumor cells. Y100 treatment disrupted proteostasis, metabolic homeostasis, and induced the formation of mitochondrial superoxide in NF1-deficient cancer cells. Previous studies also indicate that NF1/Ras-dysregulated tumors may be sensitive to modulators of oxidative and ER stress. We hypothesize that the use of Y100 and molecules with related mechanisms of action represent a feasible therapeutic strategy for targeting NF1 deficient cells.

## INTRODUCTION

The development of targeted cancer therapeutics enables clinicians to target malignancies with unique oncogenomic characteristics. However, in tumors driven by loss-of-function of a tumor suppressor, direct targeting of the driver mutation is not feasible. One example of this scenario are tumors with genomic mutation of neurofibromin 1 (*NF1*) or decreased expression of its gene product (NF1). Therefore, therapeutic studies have mainly focused on targeting loss of NF1 indirectly, focusing on the fact that NF1 serves as a Ras-GTPase activating protein (Ras-GAP). NF1 promotes Ras’ intrinsic GTPase activity and facilitates the shift of the Ras protein from an active (Ras-GTP) to an inactive (Ras-GDP) state [1]. Loss of NF1 thus results in increased active Ras, causing a concomitant increase in cellular proliferation, migration, and survival [2–4]. Heterozygous germline mutation of *NF1* results in the genetic disease neurofibromatosis type 1 [5]. Patients with neurofibromatosis type 1 can exhibit a variety of symptoms including café-au-lait spots, learning disabilities, and predisposition to tumors such as optic pathway gliomas, plexiform neurofibromas (PN), and malignant peripheral nerve sheath tumors (MPNSTs) [5, 6]. These tumors generally exhibit biallelic inactivation of *NF1* [7]. Mutation of *NF1* has also been observed in many sporadic tumor types including lung adenocarcinoma, sporadic MPNSTs, ovarian cancer, and glioblastoma (GBM) [6, 8–11]. The Cancer Genome Atlas (TCGA) studies have demonstrated that 10–23% of GBMs exhibit deletion, truncation, or missense mutation in *NF1*, with up to 38% of mesenchymal GBMs containing *NF1* mutations [11–13]. Furthermore, NF1 can be depleted in tumors by posttranscriptional regulatory mechanisms such as miRNA silencing and degradation of the NF1 protein by the proteasome [14–18]. This suggests that a subset of tumors with a wild-type *NF1* gene may not express functional NF1 protein.

Recently, progress has been made towards discovering therapeutics for the treatment of PNs and MPNSTs lacking NF1. For example, BRD4 inhibitors have shown efficacy in preclinical models of MPNST [19]. Additionally, inhibitors of the Ras effector protein MEK have been explored in preclinical studies and clinical trials for tumors linked to NF1 loss [20–25]. Finally, tumor metabolism (mTOR signaling) has been explored as a targetable mechanism in NF1-deficient tumor types using the small molecules everolimus and sirolimus [26–28]. However, these approaches have had mixed clinical success (mTOR inhibitors), still need to be demonstrated as efficacious in humans (BRD4 inhibitors), or are in the process of being evaluated in humans (MEK inhibitors), suggesting that there is still a need for novel therapeutics that target these tumors [6, 29–31].

Our group set out to discover novel small molecules to target tumors with NF1 deficiency [32]. We developed and conducted a high-throughput synthetic lethality screen to discover small molecules that selectively target yeast lacking the homolog of *NF1*, *IRA2* [32]. We identified multiple lead candidates including the previously described UC-1, an inhibitor of CTK1/CDK9 mediated processes [32]. Targeting this process appears to be effective in models of both *NF1* deficient yeast and MPNSTs, as well as in Ras-dysregulated pancreatic ductal adenocarcinoma [32, 33]. A second screen using the yeast platform also identified another lead candidate, the isoxazoloanthrone Y100, as being synthetic lethal with *ira2Δ* yeast, hereafter referred to as *nf1Δ*. In the present study, we describe the mechanisms by which Y100 inhibits cell growth and/or induces cell death in *nf1Δ* deficient yeast and NF1-deficient tumor cells.

## RESULTS

### Identification of Y100

**Figure 1:**
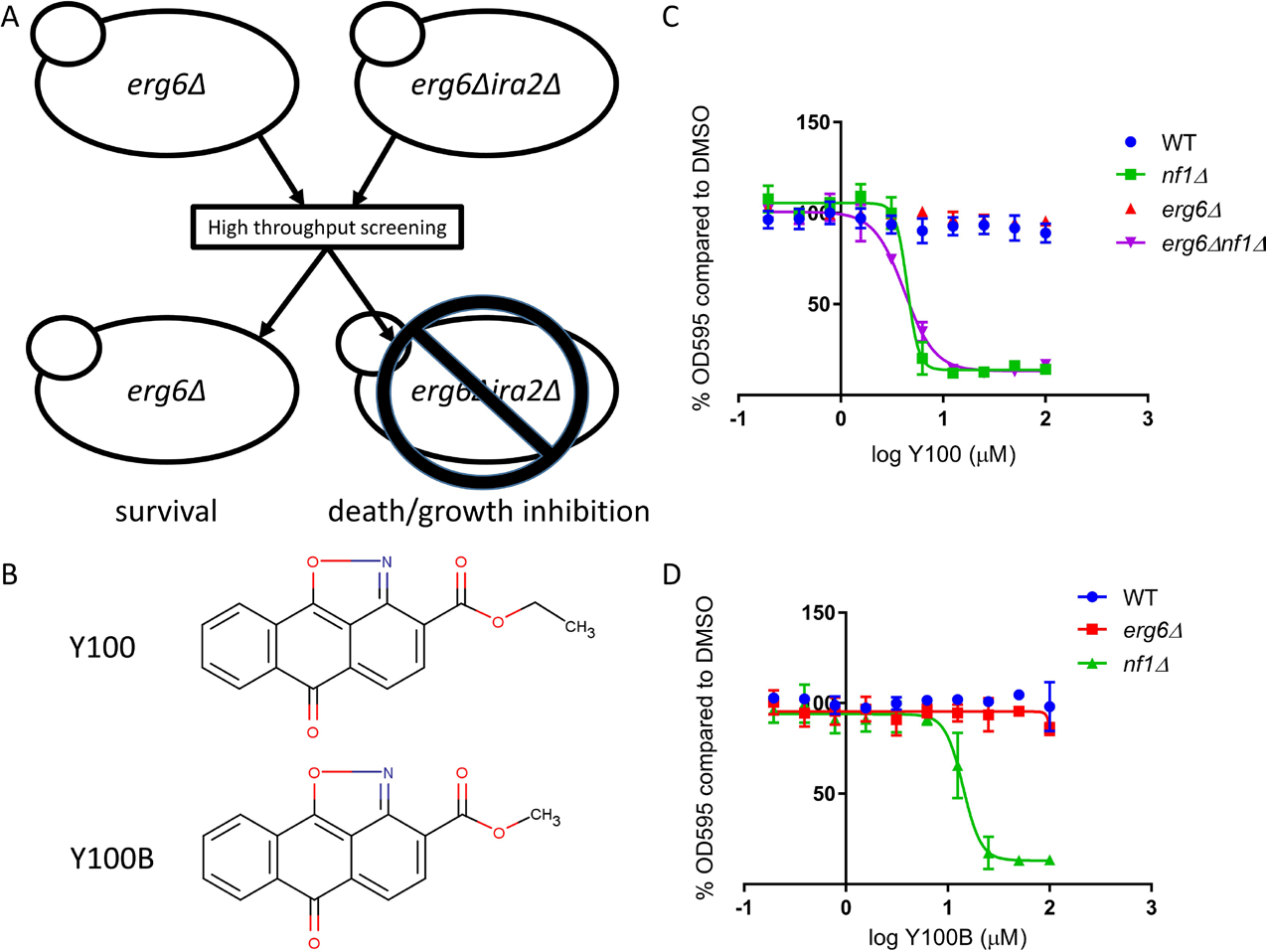
Y100 and Y100B are synthetic lethal with a yeast model of NF1 loss (**A**) A schematic of the high throughput screen for small molecules that target a yeast model of NF1 deficiency. *erg6Δ* and e*rg6Δnf1Δ* yeast were screened against ~5,100 small molecules. Cell death/growth was determined by measuring OD_600_. Small molecules that induced slow growth or death of the e*rg6Δnf1Δ* yeast without affecting the e*rg6Δ* yeast strain were considered hits. (**B**) Structure of Y100 and Y100B. (**C**–**D**) Treatment of yeast strains for 18 h with 0.2–100 μM Y100 or Y100B results in selective inhibition of *nf1Δ* strains. Control strains are unaffected. Error bars are standard deviation; graph is the average of three (Y100) or two (Y100B) independent experiments.

We conducted a high-throughput phenotypic screen to identify small molecules that selectively target cells lacking a brewer’s yeast homolog of *NF1, IRA2*. *Saccharomyces cerevisiae* lacking the Ras suppressor gene *IRA2 (nf1Δ)* were exposed to small molecules selected for structural diversity and drug-like properties (Figure 1A) [32]. The screen was conducted in *erg6Δ* strains to facilitate access of the test compounds to the yeast. Molecules that targeted *nf1Δ* cells without affecting *NF1* wild-type cells were considered “hits.” Using this approach, we previously identified a small molecule that targets a CTK1 mediated process in yeast, resulting in selective inhibition of *nf1Δ* yeast growth [32]. We subsequently screened an additional > 5,100 small molecules with this platform. In the present study, we describe efforts to identify the mechanism of action of a hit from this second screen, Y100 (Figure 1B). Y100 (CID 790239) and an analog (Y100B, CID 790235) potently and selectively inhibit growth/viability of *nf1Δ* yeast with minimal to no impact on *NF1* wild-type yeast cells (Figure 1C and 1D). The IC_50_ in *nf1Δ* yeast of Y100 was 4.46 μM, and the IC_50_ of Y100B in *nf1Δ* yeast was 14.2 μM, a difference that is likely due to the structural difference between the two molecules. The sensitivity of *nf1Δ* yeast to Y100 was not affected by deletion of *ERG6* (Figure 1C).

The mechanism by which these molecules target *nf1Δ* yeast was unknown. To identify potential mechanisms of action or targeted pathways, we performed a high copy suppressor screen. Briefly, this screen involves treating yeast containing a library of high copy plasmids that encode segments of the *S. cerevisiae* genome to identify potential “suppressors” of sensitivity to a small molecule [34, 35]. Cells overexpressing yeast genes were grown on agar containing 10 μM Y100B, vehicle (DMSO), or an unrelated small molecule identified in a previous screen (serving as a counter screen to exclude nonspecific suppressors). Colonies that survived on both vehicle and Y100B but not the counter screen molecule were isolated and the plasmids were sequenced to identify which genes were encoded in the plasmid. Highlighted genes with annotated functions are listed in Table 1, with additional genes on suppressor plasmids listed in Supplementary Table 1A [36]. The screen for suppressors of Y100 sensitivity was enriched for plasmids containing genes that encode proteins involved in autophagy or mitochondrial maintenance, among others. Gene Ontology terms were assigned to high copy suppressor screen hits to determine functional enrichment of the genes identified. 14.1% of the identified suppressors had an autophagy or vacuole-related term assigned, while 15.1% of the suppressor genes were labeled with a mitochondria-related term (Supplementary Table 1B).

**Table 1:** Selected high copy suppressor screen hits

Gene	Function
*PCP1*	Mitochondrial serine protease, homolog of human *PARL*, required for mitochondrial maintenance
*ATG23*	Membrane protein required for macroautophagy, cytosol to vacuole targeting pathway
*ATG38*	Component of autophagy-specific PIK3CA complex, required for macroautophagy
*SSK1*	Osmosensor, required for mitophagy
*PRY1*	Sterol binding, might contribute to detoxification of hydrophobic molecules
*AIM45*	May be involved in oxidative stress response
*IRS4*	Localizes Atg17p to pre-autophagosomal site, regulates autophagy
*NAR1*	Required for Fe-sulfate protein maturation, involved in oxidative stress tolerance
*CSR2*	Ubiquitin protein ligase binding protein, may regulate endocytosis

Potential suppressors of Y100 and their annotated functions.

### Y100 inhibits growth/viability and modulates proteostasis and mitochondrial health in NF1 deficient human tumor cells

**Figure 2:**
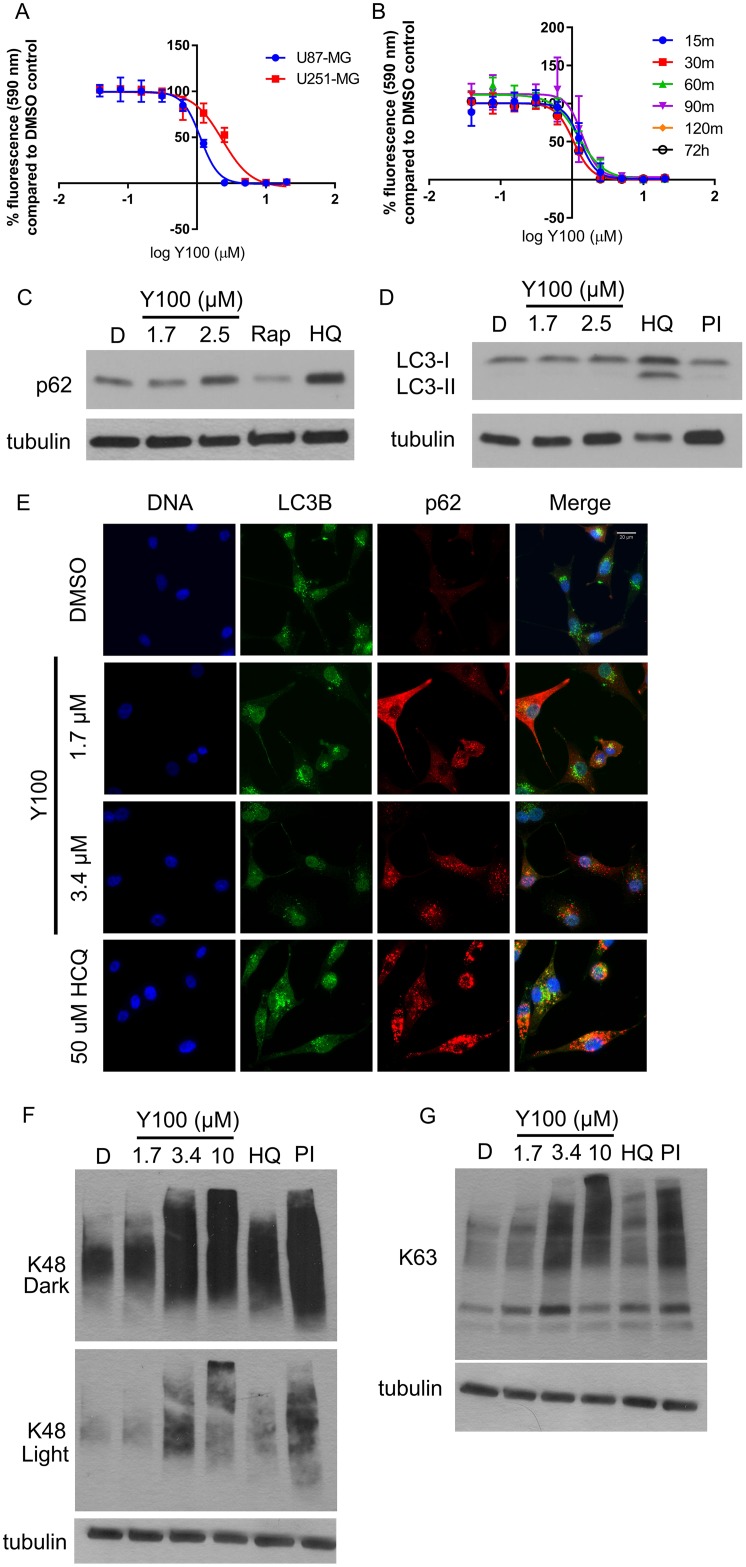
Y100 inhibits NF1 deficient mammalian cells and modulates markers of proteostasis (**A**) NF1-deficient U251-MG and U87-MG glioblastoma cells were treated with 0.039–20 μM Y100 for 72 hours. Error bars represent the standard deviation of four technical replicates. IC_50_ Y100 U87: 1.13 μM, IC_50_ Y100 U251-MG: 2.33 μM. (**B**) The effect of Y100 on cell growth/viability appears to be rapid and irreversible. U87-MG cells were treated with 0.039–20 μM Y100 for 30 minutes-72 hours. At timepoints, cells were rinsed with PBS and replaced with drug-free culture media. Data for all timepoints was collected at 72 h. Error bars represent the standard deviation of four technical replicates. (**C**) Y100 treatment induces accumulation of the autophagy marker p62. U87-MG cells were treated for 24 hours with vehicle, Y100, or the autophagy inhibitor HCQ. Cells were also treated with 2 h of MG132 and bortezomib (BTZ) as a control for proteasome inhibition. (**D**) Y100 does not result in accumulation of the lipidated form of the autophagy marker LC3 (LC3-II). U87-MG cells were treated for 24 hours with vehicle, Y100, HCQ, or the autophagy inducer/mTOR inhibitor rapamycin. (**E**) Y100 treatment induces accumulation of p62, but not LC3B. U87-MG cells were treated with vehicle, Y100 or HCQ for 24 hours and immunolabeled for LC3B (green) and p62 (red). DNA was stained with DAPI. (**F**–**G**) Y100 treatment causes the accumulation of K48- and K63-polyubiquitin linked protein. U87-MG cells were treated with DMSO (−), 1.7/3.4 μM Y100 or HCQ for 24 hours or for 2 hours with 10 μM Y100 or 1 μM BTZ/10 μM MG-132. K48/K63 linked proteins and alpha-tubulin (loading control) were detected by western blotting.

We next evaluated the effect of Y100 treatment on NF1 deficient U87-MG and U251-MG glioblastoma cells. U87-MG cells exhibit decreased NF1 due to PKC-driven proteasome-mediated degradation of the protein, while U251-MG cells have a frameshift mutation in exon 13 of one allele of *NF1*, forming a premature stop codon, and deletion of the second allele [15]. The growth/viability of both cell lines was potently inhibited with 3 days of treatment with Y100 (Figure 2A). Additionally, removal and washout of the drug within 30 minutes and subsequent evaluation at 72 hours resulted in a similar reduction in viability as a sustained 72-hour treatment, suggesting that the compound irreversibly binds its target, or that Y100’s effect is rapid and has a long duration (Figure 2B).

We evaluated apoptosis and necroptosis as potential mechanisms of Y100 induced cell death. The mechanism of action of Y100 appears to be caspase-independent (Supplementary Figure 1A). Additionally, while the effect of Y100 was abrogated by necrostatin-1 (an inhibitor of the necroptosis effector RIPK1), neither siRNA-mediated knockdown of RIPK1 nor inhibition of RIPK3 with GSK’872 was sufficient to prevent Y100-mediated cell death (Supplementary Figure 1B and 1C). Therefore, while Nec-1 inhibited the effect of Y100, the mechanism of action is likely necroptosis-independent.

The high copy suppressor screen in yeast described above suggested that Y100 might target a process involved in autophagy and/or mitochondrial maintenance. To evaluate the effect of Y100 on autophagy, we treated U87-MG cells with Y100 and evaluated the accumulation of the autophagy protein p62 as a marker of autophagy inhibition. Y100 treatment induced the accumulation of p62, suggesting increased levels of p62-linked protein and inhibition of autophagy. (Figure 2C). Unlike the autophagy inhibitor hydroxychloroquine, however, Y100 treatment does not result in accumulation of lipidated LC3B (LC3B-II), suggesting that it causes p62 accumulation via a distinct mechanism (Figure 2D). These data were recapitulated by immunofluorescent microscopy of p62 and LC3B in Y100 treated U87-MG cells (Figure 2E). Y100 also induced the accumulation of lysine 63 (K63) and lysine 48 (K48) polyubiquitin linked protein in U87-MG cells (Figure 2F–2G). K48-linked proteins are thought to be destined for proteasome-mediated degradation, while K63-linked proteins are targeted to autophagosomes [37–40]. These data suggest that Y100 treatment disrupts proteostasis, but it is unclear whether these proteins are destined for autophagic clearance, proteasome-mediated degradation, or both. One possibility is that Y100 is an inhibitor of the proteasome. An MV-151 active site probe assay suggested that Y100 is not a direct inhibitor of the proteasome and therefore is disrupting proteostasis by modulation of autophagy or another mechanism (Supplementary Figure 2).

### Y100 disrupts mitochondrial homeostasis and induces the formation of mitochondrial superoxide radicals

**Figure 3:**
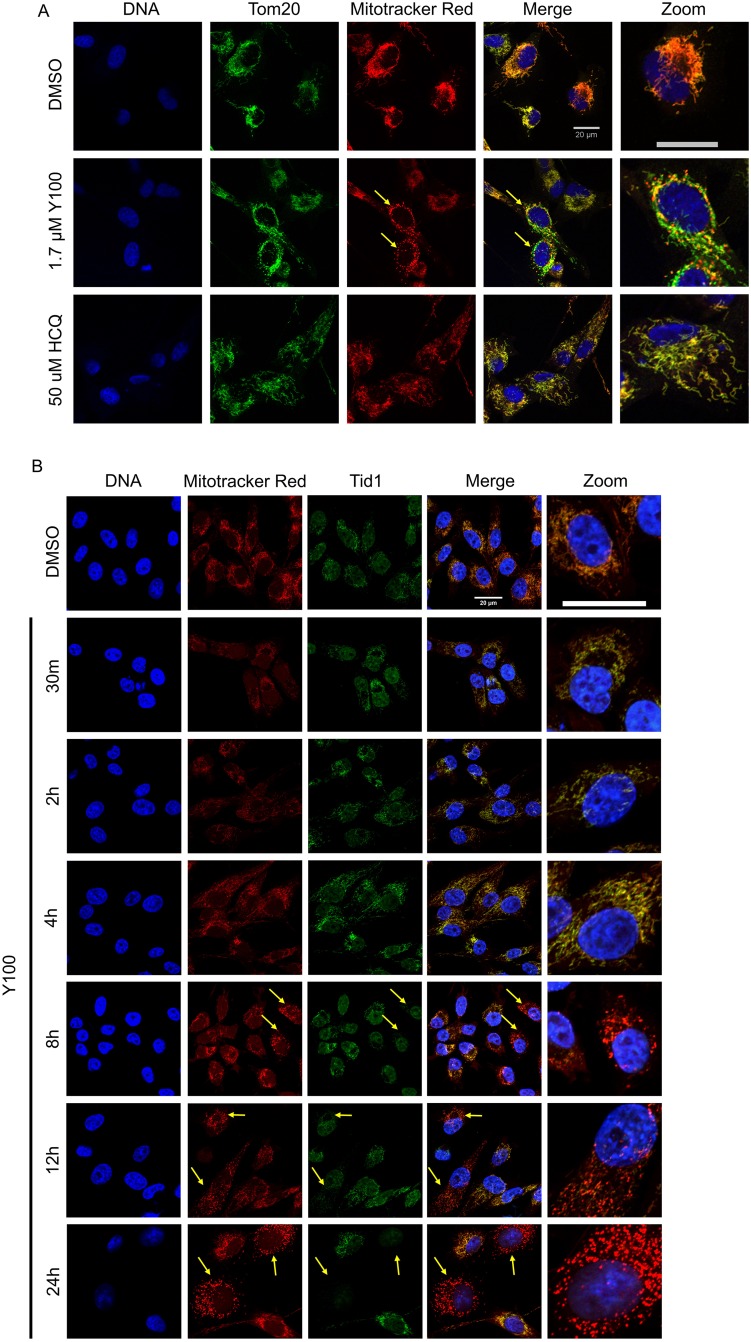
Y100 treatment causes U87-MG cells to develop polarized mitochondrial “hotspots” and cells lacking Tid1 (**A**) U87-MG cells were treated for 24 hours with vehicle, Y100 or HCQ and immunolabeled for Tom20 (green), a total mitochondrial marker, and Mitotracker (red), a marker of polarized mitochondria, and imaged on a confocal microscope. Treatment of U87-MG cells with Y100 induced the formation of polarized mitochondrial “hotspots” along the mitochondrial network (hotspot positive cells indicated with arrows). These hotspots are not observed with autophagy inhibition. (**B**) Hotspot-positive cells have reduced, or lack, Tid1, a mitochondrial heat shock chaperone protein. U87-MG cells were treated with vehicle or Y100 for 30 minutes-24 hours, immunolabeled for mitochondrial heat shock chaperone protein (Tid1, green) and polarized mitochondria (Mitotracker, red), and imaged on a wide-field microscope. Mitochondrial puncta are first observable at 8 hours of Y100 treatment. Cells with polarized mitochondrial puncta contain low to no observable Tid1 signal (indicated with arrows).

Plasmids from the screen for suppressors of Y100 also contained genes that encode proteins involved in mitochondrial maintenance and stability (Table 1, Supplementary Table 1A and 1B). We assessed the effect of Y100 on the mitochondria of mammalian cells with the mitochondrial marker Tom20 and the polarization-dependent mitochondrial marker Mitotracker Red (Figure 3A). Cells treated with Y100 had a less defined mitochondrial network (Tom20) and contained puncta of polarized mitochondria (Mitotracker Red “hotspots”) when compared to cells treated with a vehicle control. In order to confirm these findings, we examined mitochondrial polarization after Y100 treatment using the mitochondrial polarization-dependent dye JC-1 (Supplementary Figure 3). We observed the formation of polarized mitochondrial “hotspots” (red) within a broader network of depolarized mitochondria (green, Supplementary Figure 3). This “hotspot” phenotype was previously observed upon inhibition of ATP synthase with oligomycin as well as knockdown of the mitochondrial heat shock chaperone protein Tid1 [41]. We evaluated cellular Tid1 content and Mitotracker Red signal in U87-MG cells within a range of 30 minutes to 24 hours of Y100 exposure. We observed a loss of Mitotracker Red signal at shorter time points (30 minutes-2 hours) followed by the return of Mitotracker Red signal at later time points, suggesting that Y100 induced a rapid but transient depolarization of the mitochondrial network. Furthermore, Tid1 was retained for several hours after Y100 treatment but was reduced at the time points during which we observe Mitotracker Red hotspots (Figure 3B, 8–24 hours).

### Treatment with Y100 induces an oxidative stress response

To better understand the mechanism of action of Y100, we examined the transcriptional response of cells following treatment with Y100 as compared to cells treated with a vehicle control. Y100 treatment resulted in broad transcriptional changes as compared to a vehicle control (Supplementary Table 2). The transcripts with the greatest fold change are displayed in Table 2. We observed a 2.74 and 1.89 log ratio change, respectively, of *HMOX1* and *OSGIN1*/*OKL38,* which have both been identified as responsive to oxidative stress as well as regulators of the cellular response to oxidized phospholipids [42, 43]. Y100 treatment led to a 1.32 log ratio increase in *PPIF* (CypD) transcripts, an effector of the mitochondrial permeability transition that has been linked to Ras transformation [44]. The addition of a CypD inhibitor (cyclosporine A, CsA) did not prevent cell death, suggesting that although there was a significant increase in *PPIF* transcripts following Y100 treatment, PPIF protein is not required for Y100-mediated cell death (Supplementary Figure 1D).

**Table 2:** Transcripts with the greatest change upon Y100 treatment

Transcript	Log_2_ratio (Y100/DMSO)	Parametric *p*-value
*HMOX1*	2.74	0.0149326
*OSGIN1*	1.888969	0.0012831
*OKL38*	1.643856	0.0361717
*PPIF*	1.321928	0.0030423
*LAMB3*	1.286304	0.0012498
*HKDC1*	1.286304	0.0468309
*HBEGF*	1.251539	0.0013294
*SLC3A2*	1.152003	0.0290661
*CCNE2*	1.120294	0.0193412
*SLC1A5*	1.089267	0.0122129
*DHRS9*	1.089267	0.0492717
*UNKL*	1.058894	0.0130697
*HSPB8*	1.058894	0.0037441
*SPHK1*	1.029146	0.0209474
*CLDN1*	1	0.0166466
*CDK5RAP2*	1	0.0157465
*CPA4*	−2.003602237	0.0381814
*IFIT1*	−1.475084883	0.0062619
*CCNB1*	−1.384049807	0.0085255
*PLK1*	−1.304511042	0.0065993
*C13orf34*	−1.250961574	0.0181284
*HCP5*	−1.220329955	0.0109714
*CENPA*	−1.176322773	0.0140188
*KIF18A*	−1.169925001	0.006931
*AURKA*	−1.15704371	0.0016516
*HMMR*	−1.150559677	0.0246998
*CCDC85A*	−1.14404637	0.0013779
*METTL7A*	−1.111031312	0.0192033
*DLGAP5*	−1.111031312	0.0076279
*NEK2*	−1.084064265	0.0000623
*DLGAP5*	−1.070389328	0.0095592
*SYNC1*	−1.070389328	0.028098

RNA expression from Y100 treated cells was compared to vehicle treated cells. The transcript identity, log_2_ change and parametric *p*-value are listed. The ratio of gene expression and parametric *p*-value for Y100 treated cells as compared to a DMSO control is displayed.

**Figure 4:**
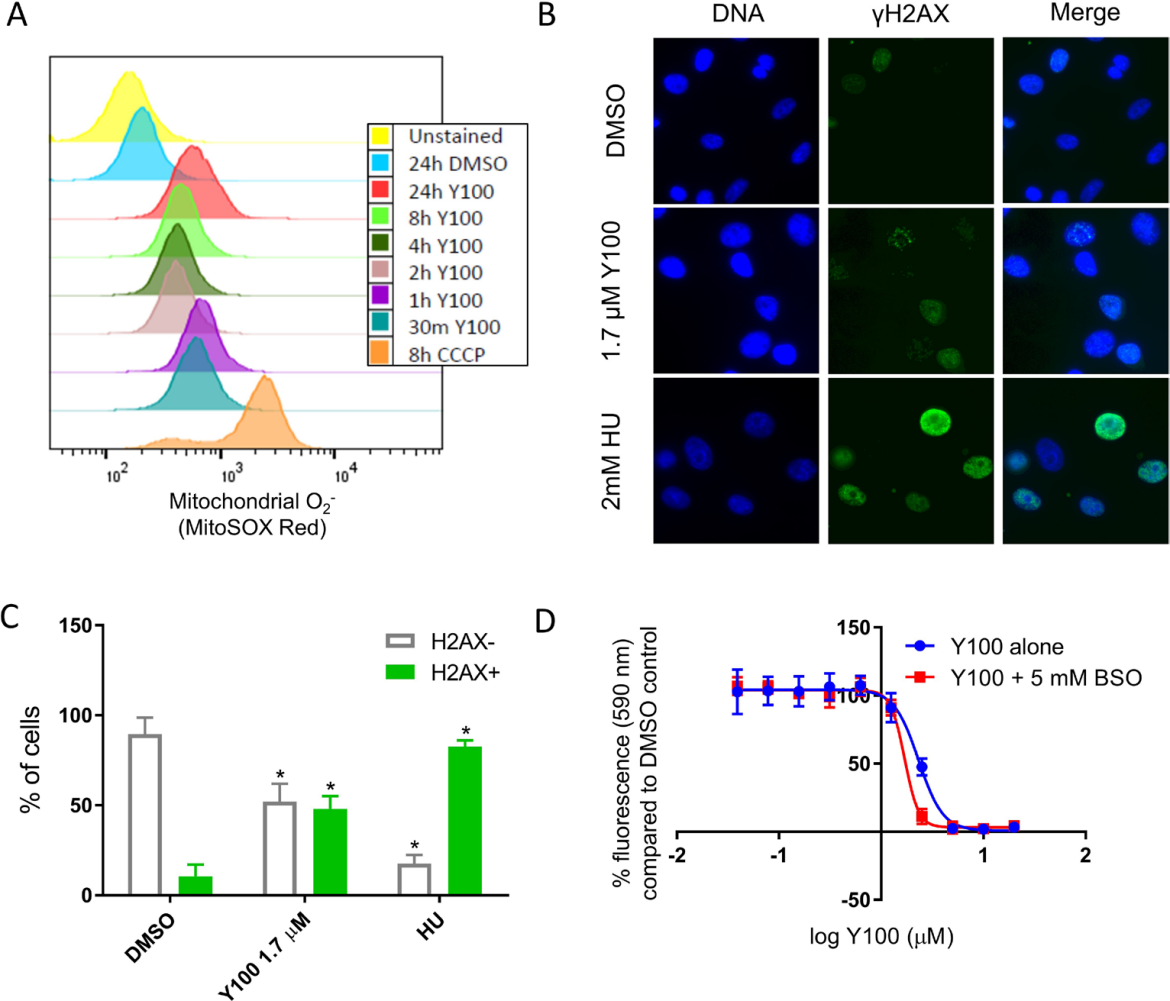
Y100 treatment causes mitochondrial superoxide and DNA damage ROS scavenging reagents abrogate the effect of Y100. (**A**) U87-MG cells were treated with vehicle, CCCP, or Y100 for a range of times from 30 minutes to 24 hours. Cells were stained with the mitochondria superoxide specific reagent MitoSOX Red, and analyzed by flow cytometry. Y100 and CCCP treatment induce mitochondrial superoxide. Y100 induced superoxide is detectable within 30 minutes of treatment. (**B**) Y100 induces DNA damage. U87-MG cells were treated for 24 hours with DMSO, Y100, or the nucleotide synthesis inhibitor hydroxyurea (HU). γH2AX (green) was immunolabeled as a marker of DNA damage, imaged with a wide-field microscope, and quantified in (**C**), where 100 cells per condition were scored as H2AX positive (3 or more nuclear foci, or pan-nuclear staining) and H2AX negative (less than three nuclear foci). Values are the average of three experiments. (*) indicates a significant difference as compared to the DMSO control, *p* < 0.005, error bars are standard error of the mean. (**D**) The GSH synthesis inhibitor buthionine sulfoximine (BSO) slightly potentiates the effect. U87-MG cells were pretreated with vehicle or BSO for 2 hours. Pretreatment media was replaced with media containing media of the same composition with 0.39–2 μM Y100 added. Cells were incubated for 72 h with drug. Viability/growth was measured with alamarBlue fluorescence.

We directly evaluated Y100 treated cells mitochondrial superoxide. Y100 treatment of U87-MG cells for 30 minutes to 24 hours resulted in the presence of superoxide radicals in the mitochondria (Figure 4A). Y100 treatment also induced DNA damage as indicated by the presence of gamma-H2AX (Figure 4B, 4C). Finally, pretreatment with the glutathione synthesis inhibitor buthionine sulfoximine moderately affected the sensitivity of U87-MG cells to Y100 (Figure 4D). Y100 treatment also resulted in a reduction of transcripts related to mitotic processes, such as *CCNB1, CENPA, PLK1, AURKA,* and *KIF18A* (Table 2). This transcriptional response may be indicative of a DNA-damage-induced cell-cycle arrest. In summary, these data suggest that Y100 induces an oxidative stress and DNA damage response, possibly a result of mitochondrial superoxide formation.

### Y100 rapidly induces oxygen consumption and altered mitochondrial bioenergetic capacity

**Figure 5:**
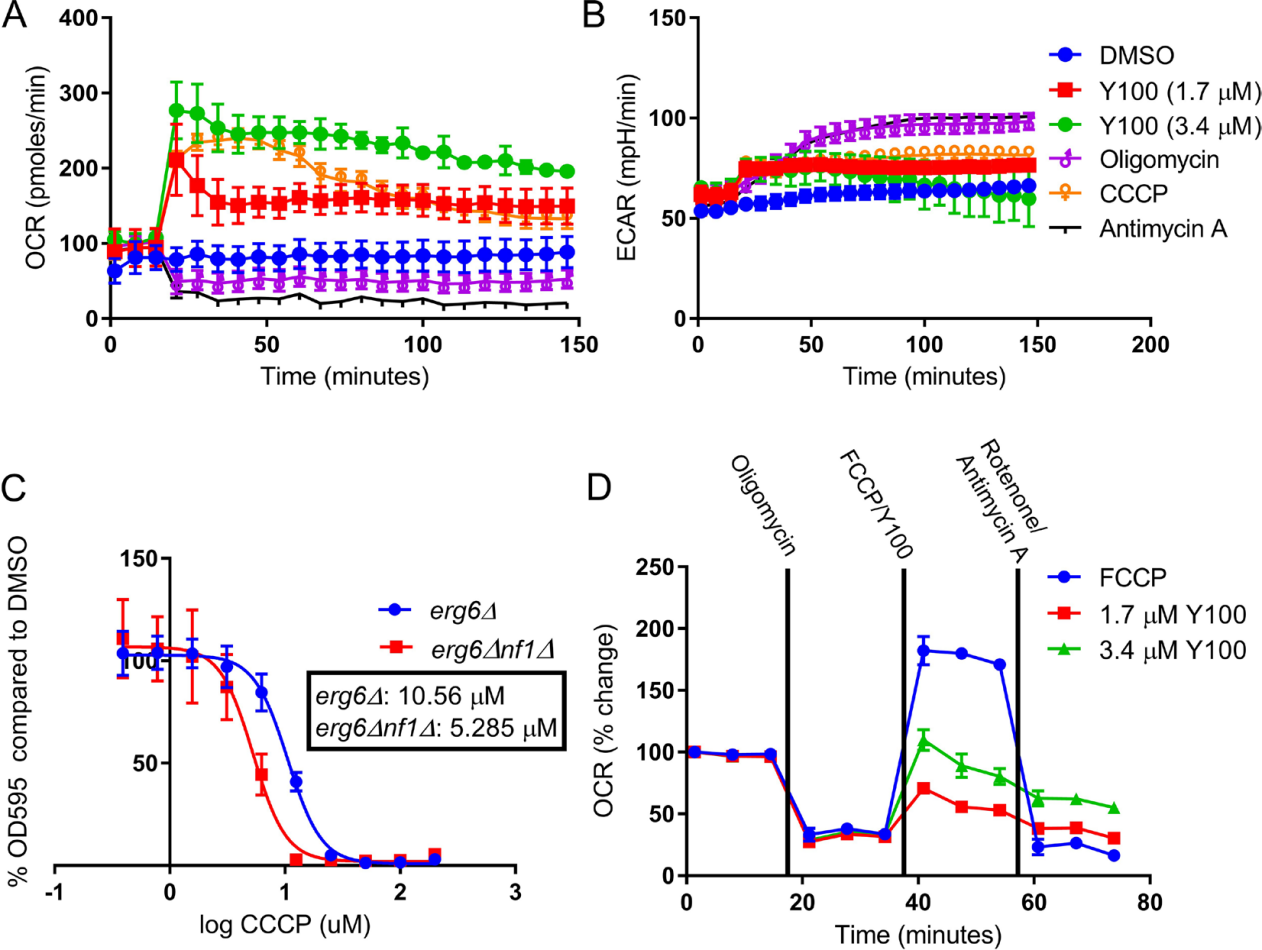
Y100 disrupts mitochondrial homeostasis (**A**–**B**) Y100 rapidly induces increased oxygen consumption, and slightly increases extracellular acidification. Metabolic function (oxygen consumption rate, OCR; extracellular acidification rate, ECAR) was measured at baseline and after injection of mitochondrially targeted agents (oligomycin, CCCP, antimycin A) as well as vehicle and Y100 (injected after 3 baseline measurements). (**C**) Cells lacking *IRA2* have increased sensitivity to the mitochondrial uncoupling agent CCCP. Yeast were treated for 18 hours in the presence of CCCP, and growth/cell death was measured with OD_600_. (**D**) Y100 increases oxygen consumption by mechanisms independent of electron transport chain uncoupling. We profiled the mitochondrial bioenergetic capacity of Y100 treated cells by performing a modified Mito Stress Test, which utilizes serial injections of oligomycin, FCCP, and a combination of antimycin A and rotenone to characterize cellular bioenergetic potential. In some samples, FCCP was substituted with Y100.

Due to the changes in mitochondrial phenotype observed after Y100 treatment, we hypothesized that Y100 treatment impacts metabolism in NF1-deficient tumor cells. To evaluate this hypothesis, we performed metabolic profiling of U87-MG cells following treatment with Y100 (Figure 5A, 5B). Three baseline measurements were taken, followed by injection of vehicle, Y100, CCCP (a protonophore that induces mitochondrial depolarization), oligomycin (an ATP synthase inhibitor), antimycin A (a cytochrome C reductase inhibitor), and bafilomycin A1 (an autophagy inhibitor). Following this injection, oxygen consumption rate (OCR) and extracellular acidification rate (ECAR) measurements were taken every six minutes for two hours. These measurements are used to evaluate oxidative phosphorylation (OXPHOS) and glycolysis, respectively. Surprisingly, the changes in OXPHOS and glycolysis upon Y100 treatment were not similar to treatment with oligomycin, which inhibited OXPHOS, increased glycolysis, and has been shown to induce the formation of mitochondrial hotspots [41]. Instead, Y100 treatment caused a rapid increase in OXPHOS comparable to treatment with CCCP. We hypothesized that NF1 deficient cells are more dependent on mitochondrial homeostasis than wild-type cells. To test this hypothesis, we treated wild-type and *nf1Δ* yeast with CCCP. We observed that CCCP is selective for *nf1Δ* yeast, suggesting that these cells are more dependent on mitochondrial homeostasis than their wild-type counterparts (Figure 5C).

Given that Y100 and CCCP both have differential activity in wild-type and *nf1Δ* yeast, and the similar metabolic profile of these molecules on NF1 deficient glioblastoma cells, we postulated that Y100 may be a mitochondrial uncoupler. To assess this, we profiled the mitochondrial bioenergetic capacity of Y100 treated cells by performing a modified Mito Stress Test, which utilizes serial injections of oligomycin, FCCP (an uncoupling agent), and a combination of antimycin A and rotenone (an electron transport chain inhibitor) to characterize cellular bioenergetic potential (Figure 5D). In order to determine the uncoupling capacity of Y100 alone, FCCP was substituted with Y100. Y100 increased OCR after oligomycin addition, but not to the same degree as FCCP. Additionally, oxygen consumption in Y100-treated cells remained elevated upon addition of rotenone and antimycin A. This finding indicates that Y100-induced oxygen consumption may in part be caused by changes to mitochondria-independent oxygen consumption, rather than mitochondrial uncoupling.

## DISCUSSION

### Model for Y100’s mechanism of action

In the present study, we demonstrate that Y100 perturbs proteostasis, mitochondrial function, and mitochondrial superoxide in NF1-deficient cancer cells and is synthetic lethal with NF1 loss in a yeast model. It may be that this molecule or others that induce a similar cellular response could be employed in a therapeutic setting to treat malignancies driven by NF1 loss.

Our working model is that Y100 has a multimodal mechanism of action resulting from its induction of mitochondrial superoxide. We observed induction of genes such as *HMOX1, OSGIN,* and *PPIF* after 8 hours of Y100 treatment, indicating that Y100 may promote an oxidative stress response and disturb mitochondrial homeostasis. Genes involved in cell division were downregulated upon treatment with Y100, suggesting that Y100-mediated toxicity may be in part due to cell cycle disruption that results from a DNA damage response or other mechanisms. We hypothesize that the superoxide generated by Y100 causes DNA damage, proteotoxicity, and cell death.

The direct molecular target of Y100 has yet to be determined. Y100-induced cell death appears to be caspase-independent, RIPK1/3-independent, and PPIF-independent. Our data suggest that Y100 treatment results in transient mitochondrial depolarization, dysregulation of mitochondrial homeostasis, and generation of mitochondrial superoxide. It may be that accumulation of p62 as well as K63 polyubiquitin-linked and K48 polyubiquitin-linked proteins is a consequence of oxidative stress, as has previously been observed [37, 45, 46].

We also observed that mitochondrial uncoupling with CCCP was more toxic to a yeast model of NF1 loss as compared to wild-type yeast. However, Y100 has a much larger therapeutic index in the yeast model than CCCP, suggesting that there could be additional mechanisms by which Y100 targets cells with NF1 loss, such as differences in ROS levels or ROS sensitivity. This mechanism could be clarified in future studies by testing the basal, CCCP, and Y100-induced ROS levels of the NF1 wild-type and deficient yeast model. Furthermore, our data showed that Y100 induced mitochondria-independent oxygen consumption, which could be the result of NADPH oxidase (NOX) pathway activation or other metabolic pathways. NOX activation is a particularly interesting candidate pathway for future studies with Y100 as NOX function has been shown to be critical for Ras-driven transformation.

### Metabolic requirements of NF1 deficient cells

Modulation of metabolism may be a worthwhile strategy for targeting NF1-deficient cells. Our data indicate that Y100 disrupts mitochondrial homeostasis, suggesting this may be one route by which Y100 targets these cells. Prior to this study, other research indicated that NF1 is localized to the mitochondria and regulates metabolism [47, 48]. More recent work by Masgras, *et al*. demonstrated that NF1 deficiency causes metabolic dysregulation (reduced respiration and increased glycolysis) that is regulated by mitochondrial ERK-TRAP1-mediated inhibition of succinate dehydrogenase and, furthermore, that NF1 dysregulated tumors require this mechanism for tumor growth [49]. These data suggest that because NF1 signaling and metabolic pathways are intrinsically linked, approaches that alter tumor metabolism may target a key vulnerability of NF1-deficient tumors. For example, NF1-deficient tumors are sensitive to the mTORC1/2 inhibitor AZD8055 [50]. In addition, it may be therapeutically beneficial to combine small molecules, such as Y100, that disrupt metabolic processes with other targeted therapies. For example, it was recently shown that the combination of mTOR inhibition and MEK inhibition had a synergistic effect in inhibiting the growth of a genetically engineered model of MPNSTs [51]. Fatty acid synthesis and lipid metabolism represents another potential targetable metabolic pathway in these tumors, as MPNST rely on fatty acid synthesis for tumor growth and cellular survival [52]. Inhibition of glutamine utilization via glutaminase inhibition has also been proposed for treatment of gliomas and NF1-deficient tumor cells [53, 54].

### Sensitivity of NF1 deficient cells to oxidative and proteotoxic stress

Tumor cells with Ras/NF1 dysregulation have been shown to be sensitive to disruption of cellular reactive oxygen species management and proteotoxicity management mechanisms. DeRaedt and colleagues have observed that tumor cells lacking NF1 were sensitive to reactive oxygen species-induced proteotoxic stress [55]. Similarly, Shaw *et al.* observed that a small molecule inducer of reactive oxygen species, lanperisone, suppressed the growth of KRAS^G12D^ mutant cells in a murine xenograft tumor model [56]. It remains to be seen whether other factors associated with NF1-deficient tumor formation such as overexpression of *PDFGRA* or loss of *TP53* and *PTEN* could act as modifiers regarding sensitivity to elevated ROS and disruption of proteostasis [23, 57, 58]. These data, as well as our own, strongly suggest that induction of oxidative and proteotoxic stress may be a tractable therapeutic approach for targeting these tumors with loss of NF1 or Ras gain-of-function mutations.

## MATERIALS AND METHODS

### Reagents

Y100 and Y100B were synthesized by Chembridge Corp (San Diego, CA). The synthesis of Y100 and Y100B was first described by Gornostaev and Lavrikova [59]. Necrostatin-1 (Nec-1) and buthionine sulfoximine (BSO) were purchased from Enzo Life Sciences (Farmingdale, NY), Q-VD-OPh hydrate (QVD) was purchased from ApexBio (Houston, TX), GSK’872 (RIPK3 inhibitor) and oligomycin (A/B/C) were purchased from Calbiochem/EMD Millipore (Billerica, MA), carbonyl cyanide m-chlorophenylhydrazone (CCCP), hydroxyurea (HU), and poly-L-lysine were purchased from Sigma-Aldrich (St. Louis, MO), antimycin A and cyclosporine A were purchased from Alfa Aesar (Haverhill, MA), and hydroxychloroquine sulfate (HCQ) was purchased from Spectrum Chemicals (New Brunswick, NJ). Stock solutions of all molecules were prepared in 100% DMSO with the exception of HCQ and HU, which were prepared in phosphate buffered saline without calcium and magnesium (Corning #21-040-CV).

### High copy suppressor screen

Yeast (MDW320, Table 3) were transformed with 1 μg of the *S. cerevisiae* AB320 genomic library in YEp13 (ATCC 37323) or empty vector (YEp13) using a transformation master mix containing 37% w/v polyethylene glycol MW 3350, 110 μM lithium acetate, and 307 μg/mL salmon sperm carrier DNA in sterile water. Cells were incubated in the transformation mixture, including DNA, for 30 minutes at 30°C before heat shock for 15 minutes at 42°C. The cells were washed once with water and then plated on freshly made SC-Leu dropout agar plates with 10 μM Y100B, DMSO, or an unrelated tool compound. Individual colonies that grew on Y100B and DMSO but not the unrelated compound plates were then grown on SC-Leu. Plasmid DNA from the surviving yeast was prepared with 25:24:1 phenol:chloroform:isoamyl alcohol and then electroporated into *E. coli.* Plasmid DNA was isolated from *E. coli* using a QiaPrep Spin MiniPrep kit (Qiagen). Plasmids were restriction digested with the restriction enzyme NdeI at 37°C for 1 hour. Cut plasmid DNA was separated on 0.8% agarose gels to identify unique plasmids. Unique targets were sequenced by the Sanger method using the following primers that recognize sequences on YEp13:

**Table 3:** Yeast strains used in the present study

Strain	Genotype	Source
MLY41a	*ura3-52*	Lorenz et. al. 1997 [60]
MDW057	As MLY41a *erg6Δ*	Wood et. al. [34]
MDW028	As MLY41a *ira2Δ*	Wood et. al. [34]
MDW035	As MLY41a *erg6Δira2Δ*	Wood et. al. [34]
MDW320	*ira2Δ leu2-3 his3-11*	This study

Forward: AAG CGC TCA TGA GCC CGA AGT G

Reverse: TCT ATG CGC ACC CGT TCT CG

Sequences were compared known yeast genes to identify high copy sequences using the Saccharomyces Genome Database WU-BLAST2 tool [60]. Functional annotation of high copy suppressor hits with Gene Ontology terms was performed with the Saccharomyces Genome Database YeastMine tool [61].

### Cell culture

U87-MG cells were purchased from ATCC (Manassas, VA). U251-MG cells were purchased from Sigma-Aldrich. U87-MG and U251-MG were passaged regularly and cultured with DMEM (Corning Life Sciences, Corning, NY) in the presence of 10% v/v fetal bovine serum (Atlanta Biologicals; Life Technologies). Immortalized mammary epithelial cells (IMECs) were a kind gift from Dr. James DiRenzo. IMECs were cultured in DMEM/F12 50/50 media supplemented with 5% FBS, 2 mM glutamine (Gibco), 5 μg/mL insulin (Akron Biotech), 500 μg/mL hydrocortisone (MP Biomedical), and 10 ng/mL recombinant human epidermal growth factor (Promega). Cells were routinely verified as mycoplasma free with the MycoProbe kit (R&D Systems).

### Immunofluorescence

50,000 U87-MG cells were cultured on poly-D-lysine coated coverslips (Neuvitro Corporation) or poly-L-lysine (Sigma) coated coverslips and treated with Y100 (Chembridge) for 24 hours. When labeling polarized mitochondria, cells were treated with 100 nM Mitotracker Red CMXRos in culture media for 30 minutes prior to fixation (Life Technologies). After treatment and labeling, cells were rinsed with PBS. Cells were fixed in methanol-free 4% paraformaldehyde, pH 6.9 (Electron Microscopy Services) prepared in PBS (Corning) for 10 minutes at room temperature (RT) and blocked with immunofluorescence buffer (2% [v/v] goat serum, 0.2% [v/v] Triton X-100 and 0.05% [w/v] sodium azide in PBS) at RT. Primary antibody conditions were diluted in IF buffer and used as follows: anti-Tom20 FL-145 (Santa Cruz, 1:200, RT, 1 hour), anti-Tid1 Ab-2 RS13 (Neomarkers, 1:200 4°C, overnight), anti-p-H2AX Ser139-488 N1-431 (BD, 1:100, 4°C, 30 minutes, no secondary). After primary antibody staining, cells were rinsed 3 times for 5 minutes with PBST. Secondary labeling was performed with 1:600 goat anti-rabbit 488 or 1:800 goat anti-mouse F(ab’)2 DyLight 594 or 647 at room temperature for 1 hour (Jackson Immunoresearch) diluted in IF buffer. Cells were then rinsed 2 times for five minutes with PBST and nuclei were labeled with 0.33 μg/mL DAPI in PBS for 5 minutes. Coverslips were mounted on glass slides with ProLong Gold (Life Technologies) and imaged. For JC-1 mitochondrial staining, PLL coated coverslips were seeded with 50,000 U87-MG cells and allowed to adhere overnight. The following day cells were treated with 10 μM CCCP, 1.7 μM Y100, or equivalent volume DMSO in DMEM with 10% FBS for 12 h. Thirty minutes before the endpoint, the medium was replaced with 1 μg/mL JC-1 dye in DMEM with 10% FBS. Live cells were then mounted in 1:1 PBS:ProLong Gold and imaged immediately. Wide-field images were acquired with a Zeiss Imager Z1 wide-field microscope equipped with a 40× 1.3 NA EC Plan-NEOFLUAR objective and Zeiss Axiovision software. Confocal images were acquired with a Nikon A1RSi confocal microscope equipped with a 60X 1.4 NA objective, a DU4 detector unit, and Nikon Elements software. Image processing was performed with Fiji, built on ImageJ2 [62, 63].

### Microarray

5 × 10^5^ immortalized mammary epithelial cells (IMECs) were cultured overnight and treated with DMSO or 1 μM Y100 for 8 hours. Cells were trypsinized, rinsed in PBS and centrifuged. The supernatant was removed and the pellet was frozen at −80°C. The RNA was extracted using a Norgen Biotek RNA/DNA/Protein Purification Plus Kit, following the standard operating protocol. 300 ng RNA/sample was labeled using the TargetAmp Nano kit (Epicentre Bio, Madison, WI). RNA was hybridized to an HT12v4 microarray (Illumina, San Diego, CA) overnight in an Illumina hybridization oven. Arrays were scanned with the iScan microarray scanner (Illumina). Analyses were performed using BRB-Array Tools Version 4.2.1. BRB-ArrayTools is an integrated software package for the analysis of DNA microarray data [64]. Variance-stabilizing transformation was applied to raw intensity data, which was then normalized using robust spline normalization and filtered to remove non-detected spots as determined by Illumina BeadStudio Software. Three technical replicates were performed for both conditions. One DMSO replicate was determined to be an outlier and thus eliminated. Differentially expressed genes were identified using a random-variance *t*-test with a *p*-value cutoff of 0.05 [64]. Multiple testing correction was performed using the method of Benjamini and Hochberg. Hierarchical clustering was employed to generate heat maps for subsets of significant genes using the open source software Cluster/Treeview written by Michael Eisen [65]. Cluster and TreeView are programs that provide a computational and graphical environment for analyzing data from DNA microarray experiments or other genomic datasets. Data were uploaded to the Gene Expression Omnibus (accession number GSE86421).

### Dose response assays

To perform drug sensitivity assays in mammalian models, cells were plated to 96-well plates at a concentration of 5,000 cells/well. After overnight incubation, the medium was removed and replaced with 100 μL of medium containing 0–20 μM Y100 and DMSO (to normalize DMSO concentrations). In the case of the Nec-1, GSK’872, cyclosporine A, and QVD co-treatment assays, cells were preincubated with these compounds or a vehicle control for 2 h before being replaced with Y100 +/− Nec-1, GSK’872, QVD, cyclosporine A, or vehicle. In the case of the BSO co-treatment assay, cells were incubated with BSO or media for 48 h before being replaced with Y100 +/− BSO. Cells were incubated for the noted time with a final 3-hour incubation in 5% AlamarBlue (Thermo Scientific). The plate was scanned using a Spectramax M2 (Molecular Devices) plate reader at an Ex/Em of 544/590 nm, and fluorescence was normalized to vehicle control wells. Each experiment was repeated 2–3 times with 4 technical replicates per experiment. A representative experiment is shown in each case.

To perform drug sensitivity assays in yeast models (Table 3), log-phase cells were diluted to 0.05 OD_600_. Drugs were diluted in SC-Complete media starting at 200 μM, followed by 9 2-fold serial dilutions to generate a 10-point range of concentrations. 75 μL of cells were mixed with 75 μL of drug dilutions in a 96-well plate, with four technical replicates per concentration. Yeast were incubated for 18 hours at 30°C. At 18 hours, the OD_600_ was read using a THERMOmax (Molecular Devices) plate reader and SOFTmax Pro 4.3 LS software. Each experiment was repeated 2–3 times with three technical replicates per experiment. OD_600_ was normalized to vehicle control wells.

Dose-response curves and IC_50_s were calculated with the Prism 6 software package (GraphPad, San Diego, CA, USA) by performing a 4-parameter logistic regression with outlier exclusion analysis.

### Flow cytometry

To determine mitochondrial superoxide levels, 500,000 U87-MG cells/well were plated in a 6-well plate and allowed to adhere overnight. The medium was then replaced with cell culture media, and DMSO, 1.7 μM Y100, or 10 μM CCCP was added to the media at the noted time points. Cells were treated for 30 minutes to 24 hours. 30 minutes before the end of the incubation, MitoSOX Red (Cell Signaling) was added to a final concentration of 1 μM. At the end of the incubation, cells were rinsed twice with PBS and trypsinized and resuspended in PBS for analysis. The cells were transferred to flow cytometry tubes and analyzed using a MacsQuant VYB 8-color flow cytometer. MitoSOX Red fluorescence was detected using the Y2-A (TdTomato) channel. 30,000 events per sample were collected and cellular debris was gated out of the dataset. This experiment was repeated three times, and a representative experiment is shown. Histograms were generated using the FlowJo or FlowLogic flow cytometry analysis software packages.

### MV-151 active site probe assay

U87-MG cells were plated (500,000 cells per well in a 6-well tissue culture plate) and allowed to adhere overnight. Cells were treated for 24 hours with vehicle control (DMSO), Y100, or a 2 h incubation of a cocktail of 1 μM bortezomib and 10 μM MG-132 (proteasome inhibitors) as a positive control. The cells were lysed with digitonin buffer (250 mM sucrose, 50 mM Tris-HCl pH 7.5, 5 mM MgCl_2_, 1 mM dithiothreitol, 2 mM adenine triphosphate, 0.2% v/v nonidet P40, 0.025% w/v digitonin). Lysates were incubated with MV-151, a fluorescent probe that binds active proteasome subunits, for 30 minutes at 37°C and samples (10 μg total protein per sample) were separated on an SDS-PAGE gel [66, 67]. The gel was then scanned on a Typhoon scanner to detect MV-151 fluorescence, and protein was transferred to a nitrocellulose membrane and probed for alpha-tubulin as a loading control. The experiment was repeated three times.

### RNA interference

U87-MG cells were plated in a 96-well plate at a concentration of 5,000 cells/well and a 6-well plate at a concentration of 1.48 × 10^5^ cells/well. Cells were transfected with siRNA targeting RIPK1 (Sigma, SASI_Hs01_00071803), or a non-targeting control (Sigma, Universal Control #1) prepared in Lipofectamine 2000 (Invitrogen) and serum-free DMEM (Corning). 96-well plates were transfected with 5 pmol siRNA/well. 6-well plates were transfected with an equivalent amount of siRNA scaled for total volume. After a 48-hour transfection period, cells were treated with Y100 for 48 hours. Dose response assay was performed as described above. Protein samples were acquired 48 and 96 hours post-transfection to verify RIP1 knockdown (Supplementary Figure 2).

### Western blotting

U87-MG cells were plated at a concentration of 500,000 cells per well in a 6-well plate and allowed to adhere overnight. Cells were treated with the noted concentrations and times of controls or Y100. After treatment, cells were harvested with trypsin, rinsed, and lysed with 75 μL modified RIPA buffer (150 mM NaCl, 1% v/v nonidet P40, 0.5% w/v sodium deoxycholate, 0.05% w/v sodium dodecyl sulfate, 50 mM Tris pH 8.0) containing 1 mM NaVO_4_, 1 mM NaF, 1 mM phenylmethylsulfonyl fluoride, 0.1 μg/mL leupeptin, 100 μM benzamidine HCl, 1 μM aprotinin, 0.1 μg/mL soybean trypsin inhibitor, 0.1 μg/mL pepstatin, and 0.1 μg/mL antipain. Protein was quantified with a bicinchoninic acid assay kit (Pierce). 30 μg of protein was prepared in 1X Laemmli sample buffer (50 mM Tris pH 6.8, 0.02% w/v bromophenol blue, 2% w/v sodium dodecyl sulfate, 10% v/v glycerol, 1% v/v beta-mercaptoethanol, 12.5 mM EDTA) and separated by sodium dodecyl sulfate-polyacrylamide gel electrophoresis on a 4–15% polyacrylamide gradient gel (Bio-Rad). Protein was transferred to a nitrocellulose membrane, blocked with 5% nonfat dry milk in TBST and probed with anti-LC3BI/II #2775 (Cell Signaling, 1:1000, overnight), anti-p62/SQSTM-1 D-3 (Santa Cruz, 1:1000, 1 h), anti-alpha-tubulin B-1-2-5 (Santa Cruz, 1:10000, 1 h), anti-K63 polyubiquitin #5921 (Cell Signaling, 1:1000, 1 h), anti-pK48 polyubiquitin #8081 (Cell Signaling, 1:2000, 1 h), anti-GAPDH ab9485-100 (Abcam, 1:1000, overnight), anti-RIP (BD Transduction Laboratories, 1:1000, overnight) in 2% milk in TBST. Secondary labeling was performed with a one-hour incubation in 1:20000 anti-rabbit HRP or 1:10000 anti-mouse HRP (Jackson Immunoresearch) diluted in 2% milk in TBST. The film was then exposed to ECL-coated blots (Pierce) and developed using a standard film processor.

### Bioenergetic characterization of Y100

Bioenergetics analysis was performed with a Seahorse XF96 Bioanalyzer (Agilent Technologies, Santa Clara, CA). Briefly, 2.0 × 10^5^ U87-MG cells were plated in DMEM with 10% fetal bovine serum, in a Seahorse XF96 Microplate and allowed to adhere overnight. A FluxPack sensor cartridge was equilibrated in XF Calibrant at 37°C overnight. Before performing the assay, cells were washed twice in standard XF Base Medium supplemented with pyruvate, glutamine, and glucose. Cells were then incubated in 180 μL XF media for 45 minutes at 37°C with atmospheric CO_2_. To perform single injection assays, 20 μL of 10X assay molecule in XF media was prepared and loaded into port A. The assay was run with 3 baseline measurements, followed by injection and 2 hours of data collection to determine changes in oxygen consumption rate (OCR) and extracellular acidification rate (ECAR). To perform the Mito Stress Test, the assay was run as described in the Mito Stress Test kit protocol with 1 μM oligomycin, 0.5 μM FCCP, and 0.5 μM rotenone/antimycin A. For curves containing Y100, FCCP was substituted with Y100. Both experiments were repeated a minimum of three times, with a representative experiment shown. The analysis was performed with Seahorse Wave 2.2/2.3.

## SUPPLEMENTARY MATERIALS FIGURES AND TABLES






