# Supplementary material for: Exploiting mitochondrial and metabolic homeostasis as a vulnerability in NF1 deficient cells
Source: Oncotarget. 2017 Jul 18;9(22):15860–75. doi: 10.18632/oncotarget.19335 (PMC5882303; doi:10.18632/oncotarget.19335)
Supplement: Supplementary file 1 [file oncotarget-09-15860-s001.pdf]

Exploiting mitochondrial and metabolic homeostasis as a vulnerability in NF1 deficient cells

SUPPLEMENTARY MATERIALS

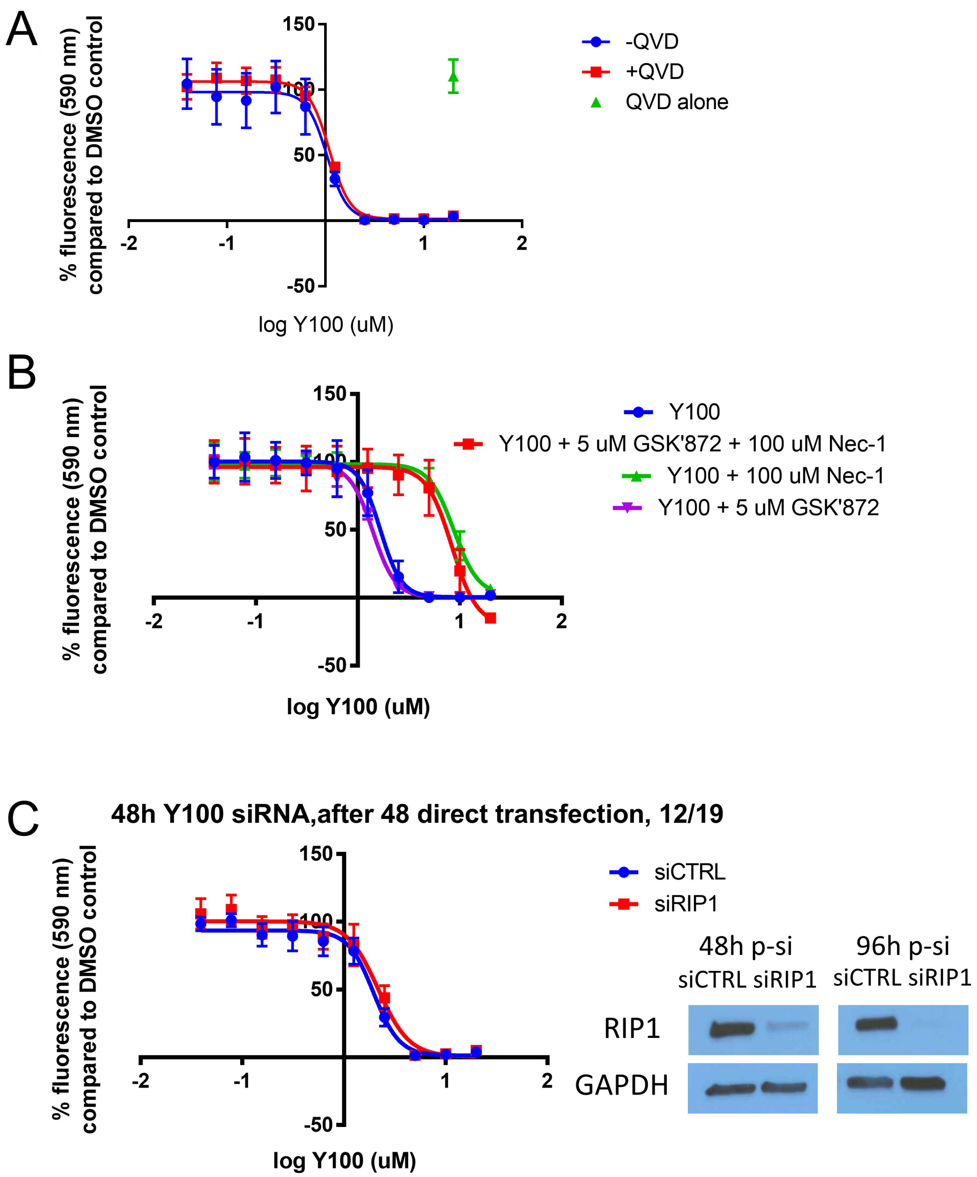

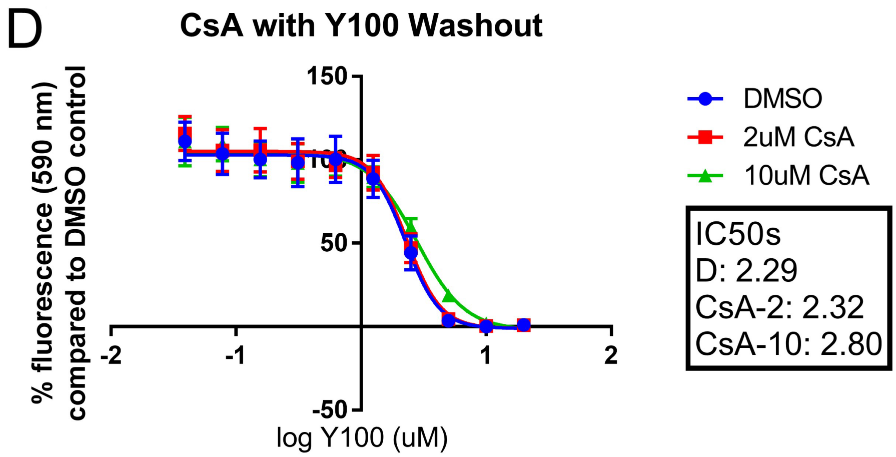

Supplementary Figure 1: Y100's mechanism of action is independent of apoptosis, necroptosis, and mitochondrial permeability transition (MPT). (**A**) U87-MG cells were pretreated with 20 µM QVD (a caspase inhibitor +QVD) or vehicle (−QVD) in media for two hours. These media were replaced with 0.39–2 µM Y100 +/− QVD, and incubated for 72 hours. Cell growth/viability was determined with alamarBlue fluorescence. (**B**) U87-MG cells were pretreated with vehicle, 100 µM Nec-1 (RIPK1 inhibitor), 5 µM GSK'872 (RIPK3 inhibitor) in media for two hours. These media were replaced with 0.39–2 µM Y100 +/−GSK'872/Nec-1, and incubated for 72 hours. Cell growth/viability was determined with alamarBlue fluorescence. Nec-1 reduces the effect of Y100, but GSK'872 does not, suggesting this may be a necroptosis-independent effect. (**C**) RIPK1 knockdown does not reduce cell death. RIPK1 was knocked down in U87-MG cells alongside a nontargeting siCTRL for 48 hours. Knockdown of RIPK1 had no effect on cell growth/viability as measured by alamarBlue (left). A western was performed to determine knockdown efficiency before (48 hours) and after treatment (96 hours) on matched but untreated samples. (**D**) Y100's mechanism appears to be MPT independent. Co-treatment with the CypD/PPIF inhibitor cyclosporine A (CsA) does not affect cell viability/growth. Cells were pretreated for 2 hours with vehicle, 2 µM or 10 µM CsA in cell culture media. This was replaced for two hours with Y100+/− CsA. Finally, after 2 hours of treatment, the media was replaced with vehicle, 2 µM or 10 µM CsA in cell culture media without Y100. Cell viability/growth was measured by alamarBlue fluorescence 72 hours after the beginning of Y100 treatment.

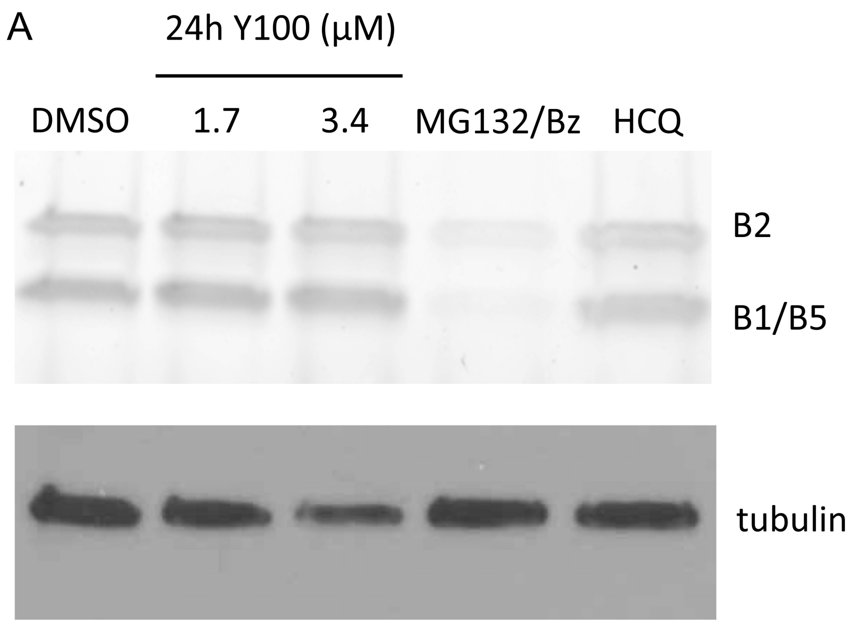

Supplementary Figure 2: Y100 does not directly inhibit the proteasome. U87-MG cells were treated with Y100, vehicle, 50 µM HCQ for 24 hours or a cocktail of 10 µM MG132/1 µM bortezomib (BTZ) for 2 hours. The cells were lysed, proteasomes were labeled with MV-151, and protein was separated with SDS-PAGE. Fluorescence was measured in-gel as a marker of proteasome activity. No change in fluorescence (indicative of proteasome activity) was observed with Y100 or HCQ. The proteins were then transferred to nitrocellulose and probed for alpha-tubulin as a loading control.

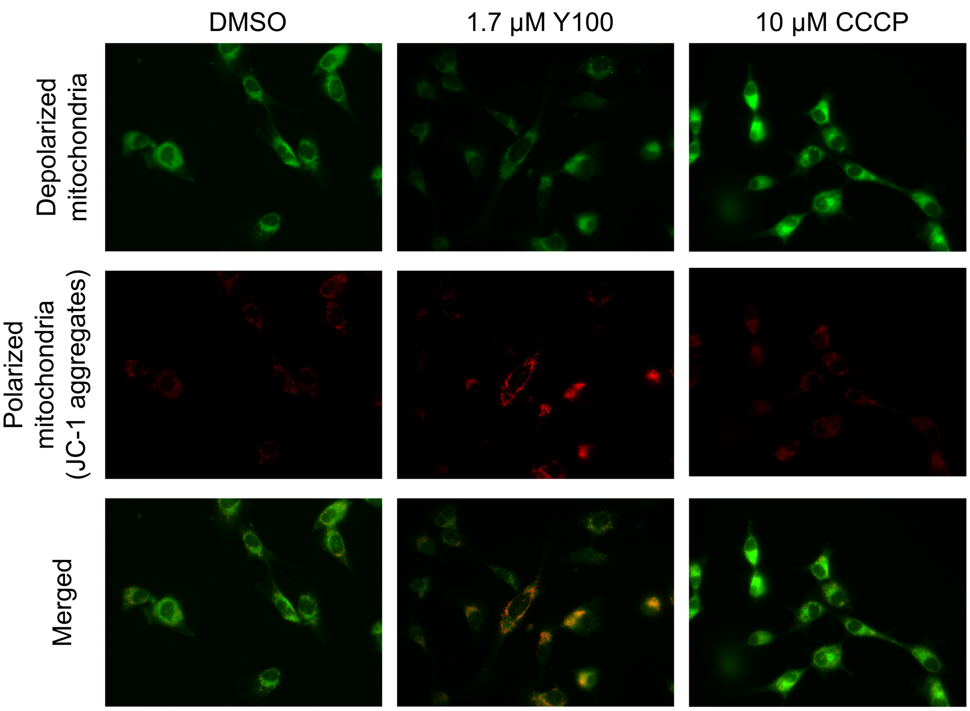

Supplementary Figure 3: Y100 treatment results in polarized mitochondrial "hotspots." Mitochondrial polarization was evaluated after Y100 treatment using the mitochondrial polarization-dependent dye JC-1. Polarized mitochondria (red) exist as "hotspots" within a network of depolarized mitochondria (green).

Supplementary Table 1A: High copy suppressor screen hits with functional annotations. See Supplementary_Table_1A

Supplementary Table 1B: Gene ontology terms associated with copy suppressor screen hits. See Supplementary_Table_1B

Supplementary Table 2: Comprehensive results table from DMSO/Y100 microarray experiment. See Supplementary_Table_2
